# Impressions from Brussels

**Published:** 1920-06-12

**Authors:** 


### Impressions from Brussels.

DR. ERIC PRITCHARD and Miss Olga Nethersole, who attended the Conference of the Royal Institution of Hygiene in Brussels, as delegates of the People's League of Health, have returned much impressed by the interest shown there in English societies for the advancement of health. The King himself gave an interview to four of the representative delegates.

In Belgium, as in England, the delegates found a sad state of apathy among the working classes in regard to health conditions. The delegates also felt that, if the Belgian Government and municipal authorities recognised the need for dissipating apathy which is responsible for much unnecessary ill-health and disease, more necessary still is the education on such matters of the people of England.

